# Initial Results From a New Model of Posterior Chamber Implantable Phakic Contact Lens: IPCL V2.0.

**Published:** 2019

**Authors:** German Roberto BIANCHI

**Affiliations:** Clínica de Ojos Dr. Nano. Centro Panamericana, Buenos Aires, Argentina.

**Keywords:** Implantable phakic contact lens, IPCL V2.0, Refractive Surgical Procedures, Cornea, Vault

## Abstract

The aim of this study was to evaluate the corneal safety, intraocular pressure (IOP), vault and refractive efficiency of the new implantable phakic contact lens, IPCL V2.0 (Care Group, India). A prospective case series study was performed to evaluate 100 consecutive surgeries with IPCL V2.0 (spherical and toric models). Refractive results, corneal endothelial cell density (CD) and central corneal thickness (CCT) were measured at baseline and 6 months after surgery. Intraocular pressure was measured at baseline, 1 day and 6 months, and vault, 3 and 6 months after surgery. Surgical complications and cataract development were also evaluated. The mean corneal endothelial CD decreased by 2.9% with a statistically significance difference (p: 0.03). The mean CCT decreased by 0.87% at 6 months postoperative, without a statistical significance difference (p: 0.35). The mean ± standard deviation (SD) of IOP at baseline was 13.72 ±1.4 mm Hg, at 1 day postoperative was 13.88 ±1.2 mm Hg, and at 6 months was 13.62 ±1.3 mm Hg. These differences were not statistically significant (p: 0.37). The difference in vault between 3 and 6 months after surgery was not statistically significant (p: 0.97). The coefficient of correlation between the attempted versus achieved spherical equivalent (SE) change was R^2^: 0.958. Postoperative SE was between -0.50 D to +0.50 D in 52% of cases. The remaining of the eyes had SE values ranging from -1.5 D to +1.35 D. No intra or postoperative complications occurred and specifically cataract was not developed. The corneal endothelial CD, CCT, vault and IOP remained stable 6 months after surgery. Refraction was improved and the IPCL V 2.0 was implanted safely.

## Introduction

In the 1990s, the laser procedures constituted a great innovation in refractive surgery [1, 2]. In addition, the cornea has demonstrated to be strong enough to resist manual intersections and incisions [3-5]. Photoablation refractive surgeries have become accessible and easy to perform for most surgeons worldwide, and these very quick procedures result in high efficiency [5]. However, as the knowledge of corneal biomechanics [6, 7]and optical aberrations [8, 9] increases, and new devices to evaluate both are now clinically available, surgeons are reconsidering refractive laser procedures to avoid complications such as postoperative ectasia, refractive regressions, high index aberration, corneal wound-healing problems and ocular surface disease, e.g. dry eye and neuropathic pain [10-14]. Refractive surgery beyond laser technology has grown through intraocular lens development. The intraocular pseudo-phakic lens has a long history, but the phakic intraocular lens (PIOL) is more recent. The design and results of PIOL are still undergoing improvement [15-21]. They could be implanted in the Anterior Chamber (AC) (iris fixated or angle supported) or at the posterior chamber. Both surgical procedures are reversible and provide maintenance of accommodation. However, this lens and its implantation procedures are associated with complications which are widely described [22-24]. Many studies have evaluated different models of PIOL (as Artisan, Artiflex, ICL) and only two papers [25, 26] exist about the implantable phakic contact lens called IPCL (Care Group, India). Nevertheless, there is a lack of scientific published information about the new model of IPCL (V 2.0). Therefore, the purpose of this work was to evaluate the safety and efficiency of implanting IPCL V2.0.

## METHODS

Study Design

A prospective case series study was performed to evaluate the safety and efficiency of 100 consecutive IPCL surgeries. This longitudinal study began in November 2017 and conducted in accordance with the Declaration of Helsinki with the approval of “Clinica de Ojos Dr. Nano” Institutional Review Board/Ethics Committee. Patients were informed about the study characteristics, their possible risks and a written informed consent was obtained before the procedures.

Inclusion/Exclusion Criteria

Inclusion criteria: Patients with myopia, when corneal refractive surgery could be contraindicated (thin corneas, severe dry eye) and, with stable refraction for a minimum period of one year.

Exclusion criteria: Patients with less than a corneal endothelial cell density (CD) 2000 cell/mm^2^; anterior chamber depth (ACD) less than 2.8 millimetres (mm); a history of glaucoma and or glaucoma or retinal surgery. In the present series, patients with hypermetropia were also excluded.

IPCL Characteristics

The IPCL is a hydrophilic acrylic, single-piece, posterior chamber PIOL. It is foldable, injectable and designed to be implanted behind the iris with the haptic zone resting on the ciliary sulcus, delivery directly through 2.8 mm corneal incision. Its design includes 6 haptics to increase stability, 2 holes in the peripheral portion from the upper zone and 4 holes outside the optical zone to facilitate their loading in the cartridge and unfolding inside the eye, placing the anterior side of the lens facing upwards. The V 2.0 has an extra central conic hole (380 micrometers [µm]) designed to minimize scattering and glare and facilitate its alignment and aqueous humour circulation. The previous iridotomy with the IPCL V2.0 is not necessary. Its dioptric power range is designed to correct myopia from -1.00 to -30.00 Diopters (D) and hypermetropia from +1.0 to +15.0 D. It was developed with an aspheric optic zone, with zero aberration. The optic diameter range is from 5.75 to 6.20 mm and the overall diameter is from 11.0 mm to 14.00 mm (with 0.25 mm steps). Moreover, the optical diameter is 6.60 mm which could be customized up to 7.25 mm, according to the pupil size of patient. Data is available from the Official Brochure of the lens, available from Care Group; (http://caregroupiol.com/products/phakic-lenses/ipcl/).

Preoperative Studies and Parameters to Evaluate

At baseline (one week preoperative), all the patients underwent a complete ophthalmic examination. The population information regarding age and gender was registered. The Pentacam imaging system (Oculus, Wetzlar, Germany) was used for preoperative evaluation of the cornea (to detect regular versus (vs.) irregular astigmatism) and to measure the ACD, which is the distance from the corneal endothelium to the anterior surface of the lens. The preoperative determination of IPCL lens size was based on the horizontal white-to-white distance (using the IOL-Master equipment). The target was emmetropia in all cases and objective (spherical and cylinder) refraction was evaluated before and 6 months after surgery. 

Preoperatively, the corneal endothelial cell density (CD) and central corneal thickness (CCT) were registered and then, 6 months after the operation, using an electronic specular microscope (TOMEY EM4000). The intraocular pressure (IOP) was evaluated at baseline, 1 day and 6 months after surgery (by Goldman tonometry). The IPCL vault was evaluated 3 and 6 months after surgery (performed with the ultra-biomicroscopy Aviso_TM_; Quantel Medical). The presence of intraoperative and/or postoperative complications was also evaluated, specifically to detect signs of cataract development (by slit lamp graded always by the same observer) according to The Lens Opacities Classification System III (LOCS III) classification. 

Descriptive statistical results were presented as mean, standard deviation (SD) and range. To compare the differences between the mean endothelial CD, CCT (baseline vs. 6 months postoperative) and vault (3 vs. 6 months after surgery), paired sample *t*-test was performed. To compare the mean IOP (at baseline, 1 day and 6 months postoperative) ANOVA (single factor) was used. A statistically significant result was considered with* a p-*value less than 0.05. The coefficient of determination (R^2^) was calculated as part of the linear regression analysis to evaluate the correlation between the attempted and achieved spherical equivalent (SE) change. Statistical analysis was performed with the XLMiner Analysis ToolPak software (Frontline Systems Inc.)

**Surgical Technique Description: Steps and Tips**

All of the surgeries were performed by the same surgeon (GB) and the use of viscoelastic substances was completely avoided. This is the routine surgical technique used by the surgeon to implant posterior chamber PIOLs and although it is not the specific objective of this study, some of its advantages were described in the discussion section. 

Steps

Under topical anesthesia, a first corneal incision (located at 45 degrees) was performed with the 20 Gauge V-lance, and the AC was maintained with infusion/irrigation cannula (a 21 Gauge bi-manual Irrigation/Aspiration (I/A) cannula). A second corneal 2.8-mm incision was performed (located 130 degrees).

Meanwhile, the AC was maintained with a balanced salt solution (BSS) fluid circulation, and the phakic lens was injected. The lens was unfolded softly with the aid of the I/A cannula, and the haptics was correctly placed behind the iris, into the sulcus, from 3 o'clock to 9 o'clock. Finally, an intracameral antibiotic (cefuroxime) was injected and the operation finished.

Tips

The BSS bottle must be elevated at 70 to 100 centimeters (cm) at all the time. A positive pressure into the AC must always be maintained and continuous BSS irrigation is required.

The IPCL was previously charged in their delivery device without viscoelastic substances. The lens is pushed to the distal part of the device, leaving it ready to be injected into the eye.

A toric lens is always placed between 0 and 180 degrees. Rotation is not needed, because astigmatism for each case came previously developed in the lens. The surgeon must be aware to mark the 0 to a 180-degree axis. The postoperative topical treatment was the same for all patients, starting three days before the operation with gatifloxacin 0.5% and bromfenac 0.09%, four times daily. Patients continued the treatment after the operation adding difluprednate 0.05% four times daily. All the drops were maintained for two weeks. 

## RESULTS

Among all of the operated cases, 8 surgeries were performed in only one eye (8 patients) and the other 92 surgeries were performed at both eyes (46 patients; one eye first and the other was operated one week later). A total of 54 patients were operated (29 women and 25 men). The mean ± SD of age was 29.7 ±7.5 years (range: 23-55). The toric models were implanted at 39 eyes and the non-toric in 61 eyes. All the surgeries were performed without intraoperative complications. Post-operative complications did not occur and 6 months after surgery, none of the eyes developed cataracts.

Corneal Safety

The mean corneal endothelial CD decreased by 2.9% with a statistically significance difference (P=0.03). The mean CCT decreased by 0.87% at 6 months after surgery, without a statistical significance difference P=0.35 (complete data is shown in Table 1).

Intraocular Pressure

The IOP values remain similar at baseline and 1 day and 6 months after surgery (Table 1). A slight increase was observed the first day post-operation, which decreased at the end of study, but the values were always in the normal IOP range. Moreover, there was no statistical significance difference between baseline, 1 day and 6 months after surgery (P=0.37). 

Vault

**Figure 1:**
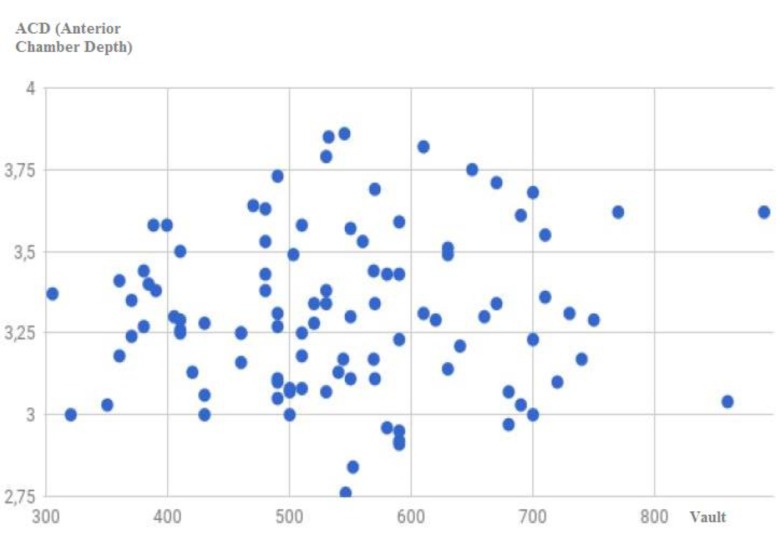
The Relation between the Vault (micrometer) and Anterior Chamber Depth (ACD) (millimeter).

**Figure 2:**
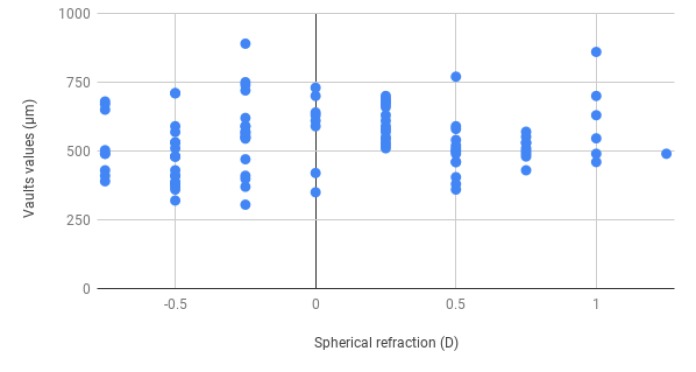
The Relation Between the Vault (micrometer) and the Spherical Equivalnt Refraction Result (D). D: diopter.

Statistical information regarding vault (3 months vs. 6 months postoperative comparison) is presented in Table 1. Results are very similar without statistically significant differences (P=0.97). Figs 1 and 2 show vaults data and their relation with ACD and the SE refraction, respectively. The preoperative mean white-to-white value was 12.24 ±0.39 mm. The mean IPCL diameter was 13.00 ±0.41 mm. The difference between white to white measurement and IPCL diameter was -0.76 ±0.20 mm. 

Efficiency: Spherical and Cylinder Refractive Results

**Figure 3:**
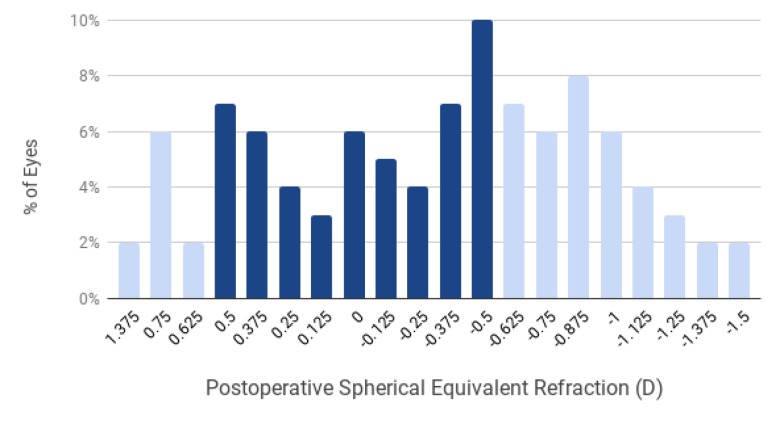
Spherical Equivalent Refractive Accuracy. The percentage (%) of eyes related to their spherical equivalent achieved at 6 months after surgery. The dark blue columns are remarking the percentage of eyes with spherical equivalent refraction between +0.50 to -0.50; summarizing 52%. D: diopter; %: percentage.

**Figure 4:**
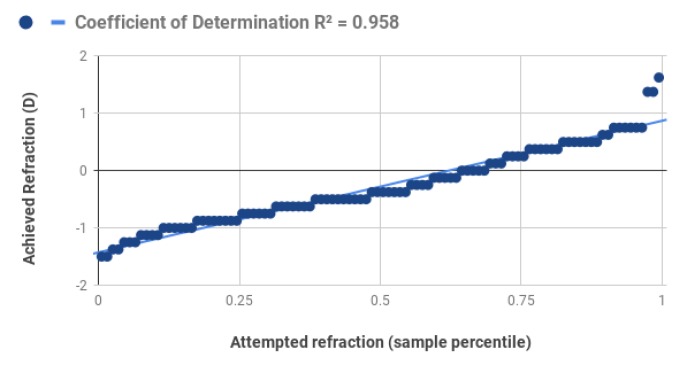
Spherical Equivalent Attempted Versus Achieved. The Correlation Between the Attempted and Achieved Spherical Equivalent (D) Change Is Shown With the Coefficient of Determination (R2). D: diopter.

Fig 3 shows the SE refractive accuracy. In total, 52% of the eyes obtained SE between -0.5 to +0.5 D. (data obtained from dark blue columns sum is shown in the Fig 3). The remaining eyes had SE values ranging from -1.5 D to +1.35 D. Fig 4 shows the coefficient of correlation (R^2^: 0.958), which denotes the strength of the correlation between the attempted and achieved SE change. The pre-operative mean ± SD (range) spherical values were -9.76 ±5.6 D (-2.25 to -22.0) and post-operative 0.04 ±0.52 D (-0.75 to +1.25). Pre-operative mean ± SD (range) cylinder values were -1.70 ±0.99 D (-4.25 to 0.00) and postoperative -0.63 ±0.25 D (-1.25 to 0.00).

**Figure 5:**
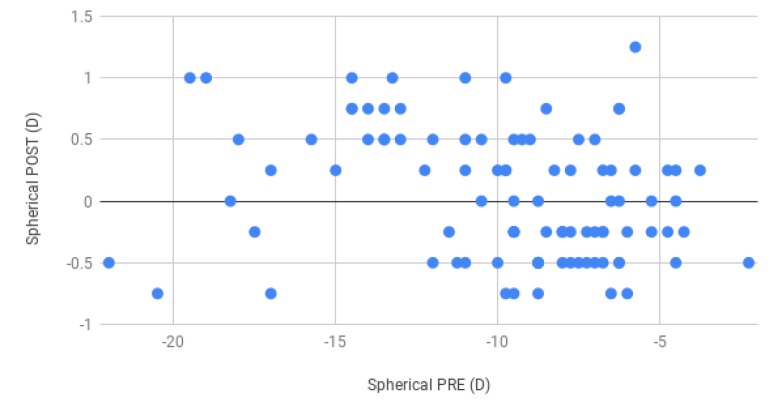
Spherical Refraction (D): Baseline Versus 6 Months Post-operative.

**Figure 6:**
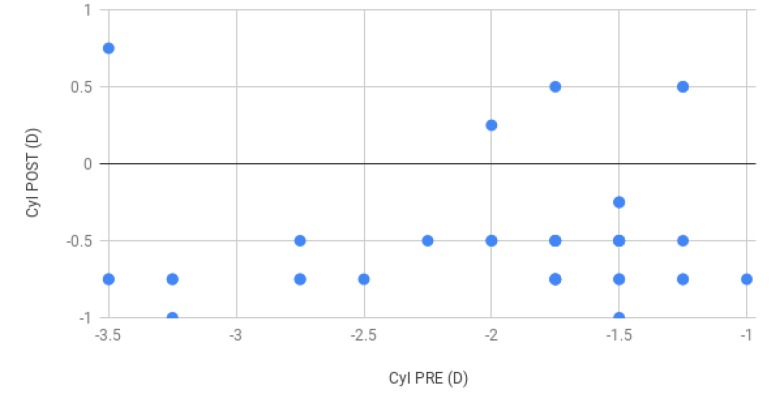
Cylinder Refraction (D): Baseline Versus 6 Months Post-operative. D: diopter; Cyl POST: postopertative cylinder result; Cyl PRE: Preoperative cylinder result.

Fig 5 and 6 show pre- and postoperative spherical and cylinder evaluation, respectively (baseline and 6 months postoperative). 

**Table 1:** The Mean Values from Endothelial Cell Density (CD), Central Corneal Thickness (CCT), Vault, and Intraocular Pressure (IOP), were presented and compared at Different Time-points.

	Baseline		Six months’ post-op		*P-*value
	Mean ± SD; (range)		Mean ± SD; (range)		
Endothelial CD (cell/mm^2^)	2625.52 ±246.18; (1870-3115)		2549.09 ±248.89; (1850-3102)		**0.03**
CCT (µm)	503.52 ±32.74; (400-576)		498.97 ±34.79; (397-595)		0.35
	**Three months post-op**		**Six months post-op**		
Vault (µm)	541.15 ±117,12; (305-890)		541.71 ±117.67; (305-890)		0.97
	**Baseline**	**One day post-op**		**Six months post-op**	
IOP (mm Hg)	13.72 ±1.4; (11-16)	13.88 ±1.2; (11-16)		13.62 ±1.3; (12-16)	0.37

**SD: standard deviation; mm** ^2^ **: square millimetre; µm: micrometer; mmHg: millimetre of mercury; Post-op: postoperative.**

P-value less than 0.05 is in bold

## DISCUSSION

The main findings of this study show that IPCL V2.0 has achieved expected refractive results, IOP and vault remained stable and corneal endothelial CD had no change 6 months after surgery. 

Refractive surface procedures using a laser are more frequently performed than intraocular procedures because it is postulated that they have fewer complications and also could be quicker and easy to perform. This is true when the right case is selected. However, obtaining independence from glasses is difficult to offer patients with high myopia or thin corneas, for whom laser corneal refractive procedures are not recommended because of the risk of complications. For this group of patients, PIOLs are a very good choice [15-21], as the present results showed.

The PIOL preserves the accommodative function. The procedure is reversible and has the advantage to induce minimal higher-order aberrations compared to corneal refractive surgeries [27]. Since their introduction in 1986, posterior chamber PIOLs have been improved considerably [28], however, to date, there are no extensive studies of the IPCL, except for only two recently published papers, with the previous model, IPCL V1.0. In one of those studies, Vasavada V et al. [25] described three years’ follow-up of 30 eyes. They reported an endothelial cell loss of 9.73%, with good refractive results and without complications. Sachdev G et al. [26] followed 134 eyes during at least one year after IPCL V1.0 model was implanted. The authors concluded that IPCL is a safe and effective procedure for correction of myopia and myopic astigmatism. The present study had many differences with those studies regarding IPCL model, IOP, endothelial CD and CCT, vault and refractive result. Some aspects of the surgical technique would be described because this is another difference with previous IPCL published studies.

The IPCL platform has had changes between V 1.0 and V 2.0 models. In the present study, the new IPCL V 2.0 model was specifically evaluated through a hundred consecutive procedures. The author of this work has a large experience and good results with the previous IPCL model V 1.0, since 2015. For this model, pre-operative iridotomy was always performed to prevent postoperative pupillary blockage and IOP rise. With IPCL V 2.0 it was not necessary to perform the iridotomy. And as seen for the results of the present series, the IOP remained stable in all cases, 1 day and 6 months after surgery. It is necessary to remark that the surgical technique for this study was performed by completely avoiding the use of viscoelastic substance. Therefore, this surgical method perhaps has an “extra value” to avoid the potential IOP peak after surgery associated with a deficiency viscoelastic extraction [28, 29].

Is it possible to work safely inside the AC without viscoelastic substance avoiding endothelial corneal damage? The author of this work has a long experience performing phacoemulsification cataract surgery without viscoelastic substance (a technique called Bianchi’s method) [30, 31]. In this work, 6 months after surgery the corneal endothelial CD decrease was 2.9%. Even though the difference was statistically significant, the procedure seems to provide very good value compared to the literature reference and there was no statistically significant difference in CCT 6 months after surgery compared to the baseline. Similar results have been published by Sachdev et al. [26] performing a surgical technique with viscoelastic substance (endothelial CD loss was 2.01% one year after surgery). This information supports the conclusion that IPCL and the present surgical technique was safe for corneal tissue. 

The posterior chamber PIOL is designed to be placed on the sulcus, behind the iris and in front of the anterior capsule of the crystalline lens. The central vault is the distance between the posterior surface of the IPCL and anterior surfaces of the crystalline lens. Several problems could arise if the IPCL size (diameter) is not correctively selected, according to the sulcus length, obtained by biometry of the eye. Because lower vault has a major risk to develop cataracts. So, the vault is an important safety parameter which also is related to refractive outcome. A secure vault value was considered to be between 250 to 750 µm and when it was close to 1000 µm the lens must be explanted [16, 24]. In this study, only 3 cases presented a vault higher than 750 µm in eyes with a deep AC (higher than 3 mm). The mean vault value observed in the present study was stable over time, without a statistically significant difference between 3 to 6 months follow-up. These data emphasize the postoperative stability and security of IPCL (as none of the cases developed cataracts). 

In the present study, the difference between white-to-white measurement and IPCL diameter was -0.76 ±0.20 mm. A correct selection of the adequate PIOL size is relevant and directly related to predicting the proper amount of PIOL vault, which is considered to be identical to the thickness of the central cornea (approximately 500 µm) in each case. Implantable phakic contact lens size changes in steps of 0.25 mm, and appropriate size is selected based on the horizontal corneal diameter and ACD. Good results may also be secondary to the “V 2.0 spring effect” due to their design. The refractive efficacy was demonstrated by the spherical and cylinder decrease after surgery. The expected refractive results have been effectively achieved. 

Another similar posterior chamber PIOL exists. Packer M. [32] has published an extensive review of literature about the Implantable Collamer Lens (ICL) with a central port called “ICL EVO V4c” (STAAR Surgical, Inc.) and he concluded that this lens is an attractive option for surgeons and patients, with high levels of refractive predictability, stability and safety results. Implantable phakic contact lens is a more recent option, with the same surgical indications as ICL EVO V4c. There is a lack of literature to compare IPCL with ICL yet and until the time of the present study, only 3 papers were published in indexed journals, about IPCL results. Further comparative studies are interesting to evaluate both lenses.

One limitation of the present work is that only one surgeon performed a particular surgical technique. Another aspect that must be considered is about the follow-up time. In the present study, results were evaluated at 6 months, which is a short time. But this is the first study reporting results with this lens and this data could be useful in future studies. Further studies with IPCL by other surgeons and longer follow-up are necessary to confirm the present results. Also, a prospective cohort multicentric study comparing the present technique with surgeries performed with viscoelastic substance is useful to confirm this specific surgical aspect. 

## CONCLUSIONS

This work evaluated IPCL results after 100 consecutive surgeries that completed 6 months follow-up, since November 2017. Regarding the surgical technique, even so, it was not the objective of this study, it was detailed described because results could be influenced by that. It is a personal technique, which could be useful for other surgeons, and this is another original aspect of this study. Findings showed that the endothelium and CCT were not affected 6 months after IPCL implantation, completely avoiding viscoelastic substances. The postoperative IOP did not increase after surgery. Vault remained stable, within safe parameters 3 and 6 months after surgery and cataract did not develop. The postoperative refraction achieved was good enough and aligned with the preoperative expectations for both spherical and cylinder (spherical and toric models). 
